# Data-Driven Multivariate Characterization of Hydrogen-Induced Response Evolution in EPDM, NBR, and FKM Elastomers

**DOI:** 10.3390/polym18131570

**Published:** 2026-06-24

**Authors:** Nitesh Subedi, Alfredo Becerril Corral, Md Monjur Hossain Bhuiyan, Omkar Gautam, Md Ariful Islam, Zahed Siddique

**Affiliations:** School of Aerospace and Mechanical Engineering, University of Oklahoma, Norman, OK 73019, USA

**Keywords:** hydrogen aging, elastomer seals, mechanical degradation, surface defects, principal component analysis, machine learning

## Abstract

Hydrogen-compatible elastomeric seals are critical for the reliability and safety of high-pressure hydrogen infrastructure. However, hydrogen exposure can alter the mechanical response and surface condition of elastomeric materials through coupled transport–mechanical interactions. This study presents a comparative experimental and data-driven investigation of the pressure-dependent degradation behavior of ethylene propylene diene monomer (EPDM), nitrile butadiene rubber (NBR), and fluorocarbon elastomer (FKM) O-ring seals following 192 h exposure to hydrogen pressures ranging from 800 to 7000 psi at room temperature. Tensile testing was performed directly on complete O-ring geometries, and descriptor-based analysis was used to quantify peak-response behavior, energy absorption, stiffness evolution, and normalized deformation characteristics. Multivariate statistical methods, principal component analysis (PCA), clustering analysis, and Random Forest regression were applied to identify material-specific degradation patterns. NBR maintained the highest overall load-bearing capability and stiffness-related response across the investigated pressure range, whereas EPDM exhibited more compliant and non-monotonic deformation behavior. FKM showed the strongest pressure sensitivity, with substantial increases in force- and stiffness-related descriptors at elevated hydrogen pressures. Optical image analysis revealed pronounced increases in defect density and defect area fraction for NBR, while FKM exhibited comparatively stable surface-state behavior. PCA and clustering analyses identified distinct material-dependent degradation trajectories, and Random Forest regression achieved an R^2^ value of 0.888 for energy-absorption prediction. The results demonstrate that hydrogen-induced degradation emerges through coupled interactions among stiffness evolution, deformation progression, energy absorption, and surface-state changes, providing a comparative framework for assessing elastomer performance in hydrogen environments.

## 1. Introduction

### 1.1. Background and Motivation

Hydrogen is increasingly regarded as a key energy carrier for enabling low-carbon energy systems across transportation, industrial processing, and power-generation applications [1,2,3]. Because of its extremely low ambient density, practical hydrogen storage and transport require compression to elevated pressures, creating significant challenges related to material durability, sealing reliability, and long-term operational safety [1,2,3,4,5]. In such systems, elastomeric components—O-rings most prominently—form the primary static and dynamic seals at fittings, valve stems, piston interfaces, and compressor heads, and their reliability directly governs hydrogen containment [4,5].

The performance of elastomeric seals in hydrogen environments depends strongly on their ability to sustain mechanical deformation while maintaining adequate contact pressure against mating surfaces [4,6]. However, exposure to high-pressure hydrogen can alter the mechanical and physical response of elastomeric materials through coupled transport–mechanical interactions involving hydrogen sorption, diffusion, swelling, and decompression-induced stress development [7,8]. These processes may produce changes in stiffness, load-bearing response, deformation stability, and surface morphology, potentially contributing to leakage, extrusion, blistering, or premature seal degradation [7,9].

In practical service environments, elastomeric seals are subjected not only to static compression but also to repeated deformation associated with cyclic pressure fluctuations, vibration, thermal variation, and mechanical actuation [10,11]. These conditions produce nonlinear mechanical phenomena, including stress softening, hysteresis, viscoelastic relaxation, and progressive microstructural evolution within the polymer network [11,12,13]. Under hydrogen exposure, these effects become further coupled with pressure-dependent transport processes, resulting in complex and potentially non-monotonic mechanical behavior [9,14].

Previous studies have shown that hydrogen-induced degradation behavior is strongly dependent on polymer chemistry, network structure, filler interactions, and pressure history [6,9,14]. Elastomer families such as ethylene propylene diene monomer (EPDM), nitrile butadiene rubber (NBR), and fluorocarbon elastomers (FKM) exhibit distinct hydrogen transport characteristics and mechanical responses due to differences in polarity, free volume, and intermolecular interactions [8,9,15]. Consequently, systematic comparison of pressure-dependent mechanical behavior across multiple elastomer systems is essential for understanding material-specific degradation trends relevant to hydrogen infrastructure applications [5,15,16].

Although substantial research has focused on hydrogen permeation, swelling, and rapid gas decompression behavior in elastomers [4,8,9], comparatively fewer studies have investigated the evolution of post-exposure tensile response using realistic sealing geometries under multiple hydrogen pressure conditions [7,14,15]. In addition, many existing investigations rely primarily on conventional mechanical metrics, such as peak force or elongation, which may not fully capture subtle changes in deformation evolution, instability behavior, or pressure-dependent response patterns [15].

Recent advances in data-driven mechanical analysis provide new opportunities for extracting additional insight from experimental force–displacement behavior [16]. Feature-extraction approaches, multivariate statistical methods, and machine-learning-assisted pattern recognition can help identify latent relationships between pressure exposure, deformation response, and material-specific behavior that may not be evident from conventional single-parameter evaluation alone [16]. Such approaches are increasingly important for interpreting complex nonlinear responses in polymeric systems subjected to coupled environmental and mechanical loading [11,12].

Accordingly, the present study investigates the comparative pressure-dependent mechanical behavior and surface degradation of EPDM, NBR, and FKM elastomeric O-ring seals following prolonged high-pressure hydrogen exposure. In addition to conventional tensile characterization, this work incorporates whole-curve mechanical metrics and multivariate data interpretation to evaluate pressure-dependent response evolution across different elastomer systems. The objective is to establish a comparative assessment approach linking mechanical response characteristics, deformation behavior, and surface degradation trends relevant to hydrogen sealing applications.

### 1.2. Knowledge Gap

Despite growing research interest in hydrogen compatibility of elastomeric materials, several important limitations remain in the current literature. Most existing studies focus primarily on hydrogen transport behavior, swelling, permeability, or decompression-induced fracture phenomena, whereas comparatively less attention has been directed toward systematic characterization of post-exposure mechanical response evolution under controlled multi-pressure hydrogen aging conditions [8,9,16]. From an engineering perspective, understanding how hydrogen exposure alters stiffness, deformation behavior, and load-bearing capability is essential because these properties directly influence contact-pressure retention, sealing effectiveness, leakage resistance, and long-term reliability of elastomeric sealing components used in hydrogen storage, compression, and distribution systems [4,5,13].

In addition, conventional evaluation approaches often rely on a limited set of scalar mechanical properties such as peak force or elongation at break [14]. While these parameters provide useful information regarding overall mechanical performance, they may not adequately capture the full nonlinear deformation behavior of elastomeric materials following hydrogen exposure. Pressure-dependent changes in force–displacement behavior and stiffness evolution are often not fully represented by conventional single-parameter analysis alone [16].

Another limitation is that many investigations employ simplified specimen geometries that do not fully represent practical sealing configurations used in hydrogen infrastructure systems. Consequently, direct correlation between laboratory measurements and engineering sealing performance remains challenging, particularly when assessing the combined influence of hydrogen exposure and mechanical loading on seal reliability [7,15].

Furthermore, although multivariate statistical analysis and machine-learning-assisted interpretation methods are increasingly used in broader materials characterization research, their application to hydrogen-exposed elastomer sealing materials remains limited [16]. In particular, comparative multivariate mechanical-feature analysis linking force–displacement response evolution, stiffness progression, deformation behavior, and degradation-state mapping across multiple elastomer systems has not been systematically established [15,16]. These limitations motivate the development of more comprehensive approaches for evaluating hydrogen-induced degradation behavior across multiple elastomer systems.

### 1.3. Objectives of This Study

The objective of the present study is to establish a comparative data-driven framework for evaluating pressure-dependent mechanical response evolution in elastomeric sealing materials following prolonged high-pressure hydrogen exposure. Commercial EPDM, NBR, and FKM O-ring seals were exposed to hydrogen pressures up to 7000 psi (≈48 MPa) under identical aging conditions and subsequently evaluated using tensile testing of complete O-ring geometries representative of practical sealing configurations used in hydrogen infrastructure systems [5,13].

Descriptor-based mechanical characterization was employed to quantify peak response, stiffness evolution, deformation progression, energy absorption behavior, and normalized force–displacement characteristics derived from the experimental tensile response [15,16]. Correlation analysis, principal component analysis (PCA), cluster analysis, and Random Forest regression were subsequently applied to identify material-specific response patterns and pressure-dependent degradation trends [15].

By combining experimental characterization with multivariate statistical and machine-learning-assisted analysis, this study provides a comparative evaluation methodology for assessing hydrogen-induced mechanical and surface-response evolution across major elastomer families relevant to high-pressure hydrogen sealing applications [13,15,16].

## 2. Materials and Methods

### 2.1. Materials

Commercial EPDM, NBR, and fluorocarbon elastomer (FKM, Viton^®^) O-rings were used as the sealing materials in this study. The O-rings were procured from McMaster-Carr (Elmhurst, IL, USA) and selected to represent elastomer systems with distinct polymer polarity, network characteristics, and hydrogen transport behavior relevant to high-pressure hydrogen sealing applications.

The EPDM and NBR O-rings corresponded to commercially available 70 Shore A sealing compounds conforming to ASTM D2000 [17] and SAE AS568 [18] specifications, while the FKM O-rings corresponded to a commercially available soft-grade Viton^®^ compound with a nominal hardness of 60 Shore A. All materials were tested using identical AS568 Dash-214 O-ring geometry to enable direct comparison of pressure-dependent mechanical response independent of geometric variation.

Each specimen was an AS568 Dash-214 O-ring with nominal dimensions of 25.0 mm inner diameter, 32.05 mm outer diameter, and a 3.53 mm cross-sectional thickness, in accordance with SAE AS568 geometry conventions. Using standardized geometry across all materials allows observed differences in mechanical response to be primarily associated with intrinsic material behavior rather than specimen dimensions.

Although the exact formulation and processing conditions of the commercial compounds were not disclosed by the manufacturers, the selected materials are representative of industrial sealing-grade elastomers commonly used in hydrogen-compatible sealing applications. EPDM, NBR, and FKM exhibit distinct polarity, intermolecular interactions, and gas-transport characteristics, making them suitable candidates for comparative evaluation of hydrogen-induced mechanical response evolution and surface degradation behavior [5,15,16]. Accordingly, the quantitative results reported in this study should be interpreted as representative of the specific commercial sealing-grade materials investigated under the present hydrogen exposure conditions, rather than as universally applicable behavior for all EPDM, NBR, or FKM formulations. Nevertheless, the selected materials provide a useful comparative basis for evaluating material-dependent response evolution among major elastomer families used in hydrogen-sealing applications.

All materials were evaluated in the as-received condition and after controlled high-pressure hydrogen exposure. The key physical and specification parameters of the elastomer O-rings used in this study are summarized in Table 1.

### 2.2. Rationale for Material Selection

EPDM, NBR, and FKM were selected because they represent commercially important elastomer families with distinct polarity, intermolecular interactions, and hydrogen-transport characteristics relevant to high-pressure hydrogen sealing applications [5,15,16]. The use of identical O-ring geometry and hydrogen-aging conditions enables direct comparison of material-dependent response evolution while minimizing geometric and experimental confounding effects. The principal hydrogen-aging conditions and tensile-testing parameters employed in this study are summarized in Table 2.

### 2.3. Hydrogen Aging Protocol

**Figure 1:**
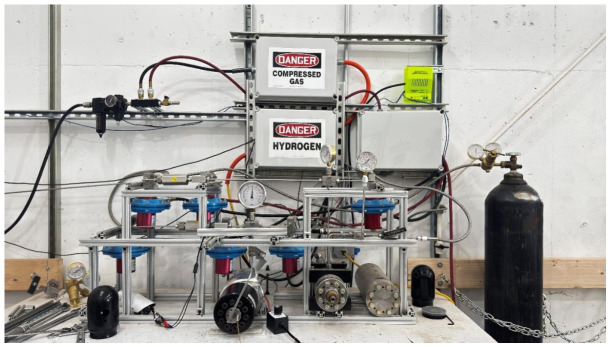
Experimental high-pressure hydrogen aging system used for elastomer exposure experiments. The setup consists of a compressed hydrogen supply cylinder, pressure regulation components, a safety monitoring system, and a GC-9 high-pressure reactor used to expose elastomer O-ring specimens to controlled hydrogen pressures under isothermal conditions. Reproduced from [15], MDPI Polymers, 2026.

Hydrogen aging experiments were conducted using the high-pressure exposure system and experimental protocol previously reported by the authors [15]. Briefly, elastomer O-ring specimens were exposed to high-purity hydrogen within a GC-9 high-pressure reactor (High Pressure Equipment Company, Erie, PA, USA) constructed from 316 stainless steel and integrated with pressure-control and safety-monitoring components. The experimental hydrogen aging system is shown in Figure 1.

Five hydrogen exposure levels spanning approximately 5.5–48 MPa (800–7000 psi) were selected to represent pressure conditions relevant to hydrogen compression, storage, and distribution infrastructure. All specimens were exposed under isothermal room-temperature conditions for a constant duration of 192 h. Following exposure, the reactor was depressurized through a fixed restriction orifice at a nominal rate of approximately 70 bar min^−1^ prior to specimen removal. The decompression rate was selected based on the operating characteristics of the available hydrogen-aging system and was maintained constant for all specimens to ensure direct comparability among materials and exposure conditions. Although decompression rate is known to influence hydrogen desorption behavior, internal stress development, and decompression-induced damage in elastomeric materials, evaluation of decompression-rate effects was beyond the scope of the present study. Accordingly, the reported trends should be interpreted within the context of the fixed decompression protocol employed herein.

Mechanical testing was initiated approximately 5–10 min after depressurization to minimize hydrogen loss prior to characterization. Consequently, the measured post-exposure mechanical response is expected to represent a near-saturated condition that may include contributions from residual dissolved hydrogen, transient plasticization, and hydrogen-induced microstructural effects. Accordingly, the measured response reflects the combined influence of residual-hydrogen effects and hydrogen-induced structural evolution, rather than either mechanism individually. Because testing was intentionally conducted shortly after depressurization, the present experimental design was not intended to distinguish between reversible plasticization effects associated with dissolved hydrogen and more persistent microstructural changes resulting from hydrogen exposure and decompression history. Additional details regarding the hydrogen aging apparatus, safety system, and operating procedure are available in Ref. [15]. It should be noted that an unexposed (0 psi) control condition was not included in this study; therefore, all comparisons are made relative to the lowest hydrogen exposure condition (800 psi).

Consequently, the primary objective of the present work was to investigate pressure-dependent response evolution among hydrogen-exposed specimens; the lowest hydrogen exposure condition (800 psi) was selected as the reference state. Consequently, the reported trends should be interpreted as relative differences among hydrogen-aged conditions rather than as absolute degradation relative to an unexposed material state.

### 2.4. Tensile Testing of O-Ring Specimens

**Figure 2:**
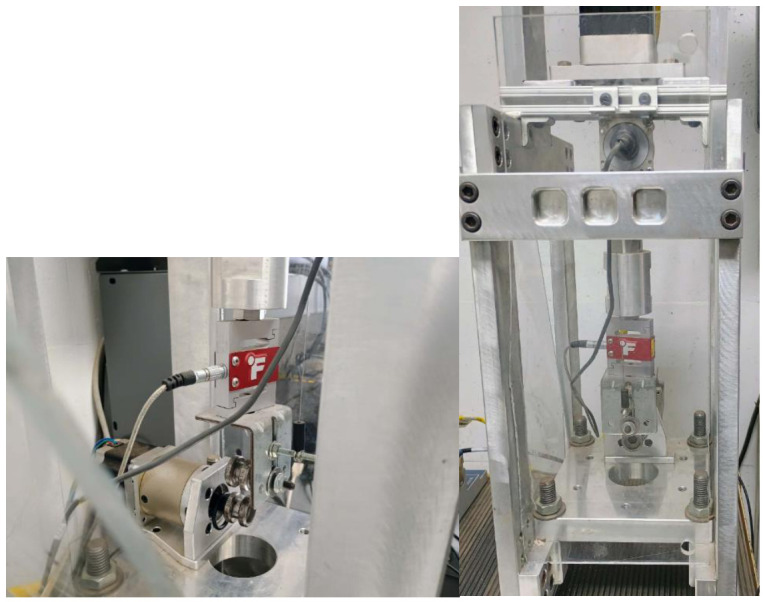
Experimental tensile testing configuration used for uniaxial loading of elastomer O-ring specimens using cylindrical spool fixtures in accordance with ASTM D1414 principles. The setup enables circumferential tensile deformation while minimizing localized stress concentrations at the grip regions. Reproduced from [15], MDPI Polymers, 2026.

Post-exposure mechanical characterization was performed using uniaxial tensile testing conducted directly on complete elastomer O-ring specimens. Tensile testing followed the general methodology described in ASTM D1414 using a spool-type loading configuration [19] and the experimental procedure previously reported by the authors [15]. In this arrangement, O-ring specimens were mounted over cylindrical spool fixtures attached to a universal testing machine, enabling circumferential tensile loading while minimizing localized gripping effects. A schematic of the spool-based tensile setup is provided in Figure 2.

Prior to testing, the spool spacing was adjusted to permit specimen installation without measurable pre-strain. Tensile loading was subsequently applied under displacement-controlled conditions at a constant crosshead rate of 0.508 mm s^−1^. Force and crosshead displacement data were continuously recorded throughout testing until specimen failure.

Because the objective of the present study is the comparative evaluation of hydrogen-induced mechanical response evolution across different elastomer systems, the analysis was based primarily on experimentally measured force–displacement behavior. The spool-type O-ring loading configuration produces a non-uniform stress field; therefore, the extracted force–displacement descriptors should be interpreted as geometry-dependent comparative response metrics rather than intrinsic material constants. However, because all specimens were tested using identical O-ring geometry, fixture configuration, and loading conditions, these descriptors provide a consistent basis for comparing relative mechanical-response evolution among materials and hydrogen exposure conditions.

In addition to conventional peak-response metrics, the recorded force–displacement curves were used to extract descriptor-based mechanical parameters including stiffness evolution, energy-related metrics, normalized response characteristics, and curve-shape-dependent features for subsequent multivariate analysis. Additional details regarding the tensile testing methodology are available in Ref. [15].

### 2.5. Mechanical Descriptor Extraction and Data Processing

Raw tensile-testing data consisted of synchronized force and displacement measurements recorded throughout loading. Prior to analysis, datasets were processed to remove preload-related artifacts, baseline offsets, signal fluctuations, and post-failure crosshead motion. Initial negative force values associated with fixture seating and preload adjustment were excluded, and data beyond the peak-force region were removed because the rapid force drop following rupture reflects specimen failure rather than continued material deformation [15]. Because the spool-based O-ring configuration does not produce a uniform stress–strain field, the mechanical response was analyzed using experimentally measured force–displacement behavior rather than conventional stress–strain quantities [15,19]. Reported displacement values correspond to the measured crosshead displacement associated with changes in spool separation during loading.

Mechanical descriptors were extracted using the framework previously developed and validated for hydrogen-exposed elastomer O-rings [15]. The descriptor set included conventional response metrics, stiffness-related parameters, normalized curve-shape descriptors, flexibility indicators, and response-evolution metrics derived from the force–displacement behavior. Table 3 summarizes the descriptors analyzed in the present study together with their physical interpretation. Mathematical definitions of the core descriptors are provided in the Supplementary Materials, while detailed implementation procedures and software workflows are available in Refs. [15,16].

Energy absorption was calculated by trapezoidal integration of the force–displacement curve from the loading origin to the displacement at peak force. Finite-deformation stiffness evolution was characterized using secant stiffness values evaluated at 25%, 50%, and 75% of the peak displacement (K25, K50, and K75) through interpolation-based analysis. Compared with differential stiffness estimation, the secant-based approach provides greater robustness against local signal fluctuations.

To compare deformation behavior independently of absolute force magnitude, force and displacement histories were normalized by their respective peak values prior to rupture and interpolated onto a common displacement grid [15,16].

For each condition, arithmetic mean, standard deviation, and coefficient of variation were calculated from replicate measurements. Reported values are presented as mean ± standard deviation. All data processing, descriptor extraction, normalization, statistical analysis, figure generation, and optical image analysis were performed using custom Python-based (version 3.13) workflows employing NumPy 1.26.4, Pandas 2.2.2, SciPy 1.13.1, Scikit-learn 1.5.1, and Matplotlib 3.9.2 [19,20,21,22].

### 2.6. Multivariate Statistical and Machine Learning Analysis

To investigate latent relationships between hydrogen exposure conditions, material type, and mechanical response evolution, multivariate statistical analysis and machine-learning-assisted interpretation methods were applied to the extracted mechanical descriptor dataset.

Correlation analysis was first performed to evaluate relationships among mechanical descriptors and to identify coupled response behavior associated with stiffness evolution, deformation progression, and energy absorption characteristics. Principal component analysis (PCA) was subsequently employed as an exploratory dimensionality-reduction method to visualize dominant patterns in the standardized descriptor space across different elastomer systems and hydrogen exposure conditions [22,23].

To further examine similarities and differences among the investigated elastomer responses, unsupervised clustering analysis was performed using the normalized mechanical descriptor dataset. To determine the appropriate number of clusters, k-means clustering solutions with k values ranging from 2 to 5 were evaluated using the average silhouette coefficient as a validation metric. The highest silhouette score was obtained for k = 3 (0.625), compared with 0.483, 0.599, and 0.458 for k = 2, 4, and 5, respectively. Because k = 3 provided the best balance between cluster compactness and cluster separation, this value was selected for the final clustering analysis.

The objective was to determine whether specimens exhibiting similar multivariate mechanical characteristics naturally group together in descriptor space without using material labels during the clustering process. This approach provides an additional means of visualizing material-dependent response patterns and pressure-associated variations that may not be apparent from individual mechanical descriptors alone.

Comparative regression analysis was additionally performed using ensemble machine-learning methods to examine relationships between extracted descriptors and pressure-dependent degradation behavior. Feature-importance analysis was used to identify the most influential descriptors governing comparative mechanical response evolution across the investigated elastomer systems [24].

Random Forest regression was selected over linear regression because the relationship between mechanical descriptors and hydrogen pressure was expected to be nonlinear and material-dependent, as anticipated from the literature on coupled sorption–decompression behavior in elastomers [24]. Given the relatively small dataset size (n = 57 specimens across the three materials and five hydrogen pressure conditions), the model was trained using leave-one-out cross-validation (LOOCV), in which each specimen was sequentially held out as a test point while the remaining specimens served as the training set [23]. Model hyperparameters were kept at scikit-learn defaults (n_estimators = 100, max_depth = None, min_samples_split = 2) to avoid overfitting through tuning on a small sample. Model performance was assessed using the coefficient of determination (R^2^) and root-mean-square error (RMSE) on held-out predictions. Feature importance was estimated from the mean decrease in impurity across the forest. The intent of this analysis is not to deliver a generalized predictive model—the dataset is too small for that claim—but rather to identify which descriptors contribute most strongly to the observed differences in mechanical response across the investigated elastomer systems under the present experimental conditions, thereby providing a data-driven complement to the descriptor-based statistical analysis.

The applicability domain of the Random Forest model is limited to the investigated experimental space, consisting of the three commercial elastomer systems (EPDM, NBR, and FKM), the selected hydrogen exposure pressures (800–7000 psi), the 192 h aging duration, and the fixed decompression protocol employed in this study. Consequently, the model functions as a comparative within-dataset analysis approach rather than as a generalized predictive tool applicable to arbitrary elastomer formulations, hydrogen exposure histories, or service environments.

A comparative composite hydrogen response index (CHRI) was additionally introduced to provide a normalized, multi-descriptor representation of the pressure-dependent mechanical and optical response evolution across the investigated elastomer systems. The index was calculated using standardized descriptor contributions according to:

$$\mathrm{CHRI}=\frac{|Z(\Delta K50)|+Z(\mathrm{Defect}\;\mathrm{Density})+Z(\mathrm{Area}\;\mathrm{Fraction})}{3}$$

where *Z* denotes z-score normalization across the experimental dataset and ΔK50 represents the deviation of the secant stiffness at 50% peak displacement relative to the corresponding 800 psi reference condition for each material. The three descriptors were selected as representative single-variable summaries of the three response domains analyzed in this work: mechanical stiffness evolution (K50), surface defect frequency (defect density), and surface defect spatial extent (area fraction). K50 was selected from the secant stiffness family because it is evaluated over a sufficiently large portion of the loading curve to reduce sensitivity to fixture seating effects while remaining within the deformation regime relevant to sealing response prior to rupture. Defect density and area fraction were retained as complementary optical descriptors that, although partially correlated, capture different geometric characteristics of the surface state. Defect density reflects the frequency of localized surface features, whereas area fraction reflects the total spatial coverage of segmented defect regions. The P90 defect-size metric was excluded from the index because it exhibited comparatively non-monotonic and redistribution-sensitive behavior across pressure conditions that did not consistently contribute to a directional response indicator.

Equal weighting was adopted because no experimentally validated calibration presently exists linking the individual descriptors directly to seal leakage, sealing failure, or service lifetime under hydrogen exposure conditions. Consequently, the CHRI is intended solely as a comparative within-dataset response indicator, rather than as a calibrated engineering failure criterion or predictive lifetime metric. The primary value of the CHRI is therefore not prediction, but dimensionality reduction, providing a concise representation of the combined mechanical and optical response state that facilitates comparison of pressure-dependent response evolution among different elastomer systems within a common normalized framework.

Because of the relatively limited dataset size and experimentally constrained pressure conditions, the machine-learning-assisted analysis should be interpreted primarily as a comparative, exploratory assessment approach rather than as a statistically generalized predictive model suitable for extrapolation beyond the investigated materials and conditions.

## 3. Results and Discussion

The results are presented using descriptor-based mechanical analysis, multivariate statistical and machine-learning-assisted interpretation, and complementary optical surface characterization to evaluate pressure-dependent hydrogen-induced response evolution across EPDM, NBR, and FKM elastomers. The emphasis is placed on identifying comparative material-dependent response patterns rather than assigning definitive molecular-level degradation mechanisms.

### 3.1. Pressure-Dependent Evolution of Peak Mechanical Response

**Figure 3:**
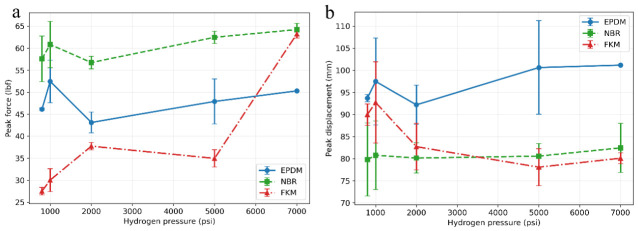
(**a**) Peak force and (**b**) displacement at peak force for EPDM, NBR, and FKM O-ring specimens following hydrogen exposure at different pressures. Values represent the mean ± standard deviation obtained from replicate tensile tests. Connecting lines are included only to guide visualization of the overall pressure-dependent response and do not imply a continuous or monotonic physical relationship between adjacent pressure conditions.

The pressure-dependent evolution of peak force and displacement at peak force was first evaluated because these parameters are commonly used descriptors of elastomer tensile behavior. Figure 3 presents these quantities side by side, enabling direct comparison of load-bearing capability and pre-rupture deformation capacity across EPDM, NBR, and FKM.

The peak force data in Figure 3 reveal clear separation among the three elastomer systems. NBR sustained the highest load-bearing response across the investigated conditions, whereas EPDM consistently exhibited lower peak-force values, reflecting a more compliant tensile response. FKM exhibited the strongest overall pressure sensitivity among the investigated materials. Although variability was observed at several pressure conditions, the mean peak-force values at the highest exposure pressure were greater than those measured at the lower-pressure conditions, indicating a stronger pressure-dependent redistribution of mechanical response compared with EPDM and NBR. Because several pressure levels exhibited substantial specimen-to-specimen variation, the intermediate fluctuations should be interpreted as part of an overall nonmonotonic response rather than as evidence of discrete mechanical transitions between adjacent pressure conditions.

The displacement corresponding to peak force also exhibited material-dependent pressure-response behavior. EPDM showed the largest displacement values across most exposure conditions, indicating greater deformation capacity prior to rupture. NBR displayed relatively stable displacement behavior with limited pressure sensitivity, while FKM exhibited pressure-dependent variation in displacement at peak force. However, the relatively large standard deviations observed at several pressure levels indicate that the response was not strictly monotonic. Consequently, the results are more appropriately interpreted as evidence of pressure-sensitive deformation behavior rather than as distinct sequential reductions and recoveries between adjacent pressure conditions.

**Figure 4:**
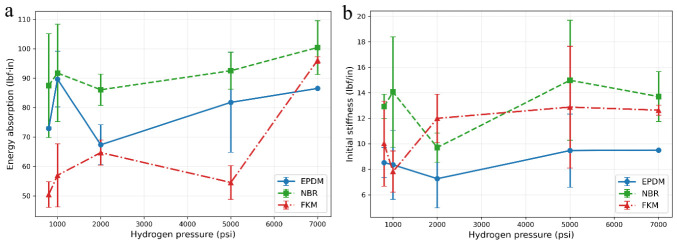
Pressure-dependent evolution of (**a**) energy absorption and (**b**) initial stiffness for EPDM, NBR, and FKM O-ring specimens following 192 h hydrogen exposure at room temperature. Error bars represent the mean ± standard deviation from replicate tensile tests. Energy absorption was calculated from the area under the force–displacement curve prior to failure, while initial stiffness was determined from the early-stage linear response of the loading curve. Connecting lines are provided solely as visual guides to illustrate overall response tendencies across pressure conditions.

Taken together, the peak-force and displacement results demonstrate that hydrogen exposure influences both load-bearing capability and deformation behavior, with the magnitude and pressure dependence of these effects varying among the investigated elastomer systems. However, peak-response parameters alone do not fully describe the response evolution observed throughout the force-displacement curves. To provide a more comprehensive assessment of deformation behavior, Figure 4 examines two whole-curve descriptors—energy absorption and initial stiffness—that quantify the work absorbed prior to failure and the resistance to deformation during the early stages of loading, respectively.

Figure 4a shows that NBR consistently exhibited the highest energy absorption across the investigated pressure range, indicating a greater capacity to sustain combined loading and deformation prior to rupture. The absorbed energy remained relatively stable at intermediate pressures and increased further at higher hydrogen pressures, reaching its highest values near 7000 psi. This behavior suggests that NBR retained a substantial ability to accommodate deformation while maintaining mechanical integrity throughout the investigated exposure conditions.

EPDM displayed intermediate energy-absorption behavior with a generally non-monotonic response across the investigated pressure range. Although differences in mean values were observed among pressure conditions, the relatively large standard deviations indicated substantial variability within several groups. Accordingly, the results suggest pressure-dependent response redistribution rather than a clearly defined monotonic increase or decrease with hydrogen pressure. The relatively large standard deviations observed for EPDM at several pressure levels indicate greater variability in post-exposure mechanical response, possibly associated with heterogeneous deformation behavior within the material.

FKM generally exhibited lower energy-absorption values at the lower pressure conditions and higher mean values at the highest investigated pressure. However, because variability was present across several exposure conditions, the results are interpreted as evidence of overall pressure sensitivity rather than a discrete transition occurring at a specific pressure level. This behavior is consistent with the simultaneous increase in peak force observed previously in Figure 3 and suggests a transition toward increased resistance to deformation at elevated hydrogen pressure conditions. FKM exhibited higher mean energy-absorption and force-related response at the highest investigated hydrogen pressure, indicating greater pressure sensitivity than EPDM and NBR under the present experimental conditions. However, the current dataset does not permit direct identification of the structural or molecular mechanisms responsible for this behavior because the study was limited to mechanical characterization and optical surface analysis. Consequently, the observed response should be interpreted as a pressure-dependent mechanical trend rather than as evidence of a specific hydrogen-induced structural transformation. Complementary techniques such as SEM, FTIR, DMA, hydrogen sorption measurements, and free-volume characterization would be required to establish the underlying structure-property relationships.

Figure 4b presents the evolution of initial stiffness as a function of hydrogen pressure. Compared with energy absorption, the stiffness trends exhibited less overall variation but still revealed distinct material-dependent behavior. NBR generally maintained the highest stiffness values across the investigated conditions, indicating greater resistance to initial deformation during loading. EPDM consistently exhibited the lowest stiffness values, corresponding to its softer deformation response. In contrast, FKM generally exhibited higher mean stiffness values at the elevated pressure conditions than at the lower-pressure conditions, indicating greater pressure sensitivity than observed for EPDM or NBR. Given the observed variability, the data support an overall increase in stiffness-related response with pressure rather than a strictly progressive pressure-by-pressure increase.

Together, the energy-absorption and stiffness results demonstrate that material differences persist throughout the loading process and are not limited to peak-response behavior alone. Because initial stiffness reflects only the earliest stages of deformation, additional stiffness descriptors evaluated over larger portions of the loading curve were examined to determine whether these material-dependent differences remain consistent as deformation progresses.

**Figure 5:**
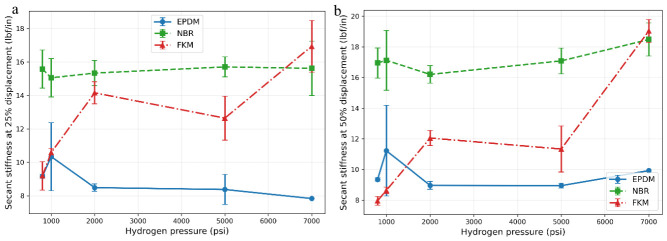
Pressure-dependent evolution of secant stiffness measured at (**a**) 25% of peak displacement (K25) and (**b**) 50% of peak displacement (K50) for EPDM, NBR, and FKM O-ring specimens following 192 h hydrogen exposure at room temperature. Error bars represent the mean ± standard deviation from replicate tensile tests. Connecting lines are included only to facilitate visualization of overall trends and should not be interpreted as evidence of continuous pressure-dependent transitions between neighboring data points.

Initial stiffness is dominated by seating effects and reflects only the first ~5% of the loading record. To follow how each material redistributes load as deformation proceeds, Figure 5 plots secant stiffness evaluated at 25% and 50% of the displacement at peak force (K25 and K50). Because these descriptors are evaluated over substantially larger portions of the loading curve, they are less sensitive to initial transients and provide a more stable view of mid-loading mechanical behavior.

The K25 and K50 descriptors largely reproduce the material ordering established previously in Figure 4, confirming that the relative stiffness differences among EPDM, NBR, and FKM persist beyond the initial loading regime. The similarity between the K25 and K50 trends indicates that hydrogen-induced response evolution affects not only the earliest stages of deformation but also the intermediate stages of tensile loading. The stronger separation observed in K50 further suggests that material-dependent differences become more pronounced as deformation progresses, highlighting the importance of evaluating stiffness evolution over multiple stages of deformation rather than relying solely on initial-loading behavior. It should be noted that several pressure conditions exhibited appreciable variability, as reflected by the standard deviations. Therefore, the secant-stiffness descriptors are interpreted primarily in terms of overall material-dependent response tendencies rather than as evidence of abrupt mechanical transitions between neighboring pressure levels.

**Figure 6:**
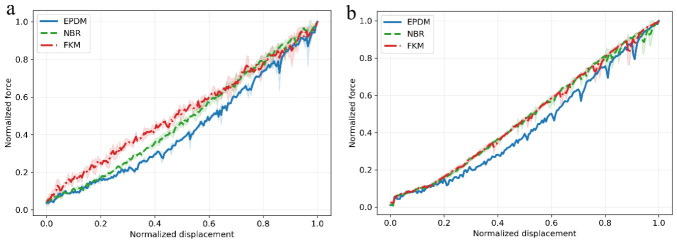
Normalized mean force–displacement responses of EPDM, NBR, and FKM O-ring specimens following hydrogen exposure at (**a**) 800 psi and (**b**) 7000 psi. Force and displacement were normalized by their respective peak values to enable comparison of relative deformation behavior, independent of absolute mechanical magnitude. Shaded regions represent mean ± standard deviation from replicate tensile tests.

Overall, the secant stiffness descriptors confirm that the three elastomers differ not only in peak-response behavior but also in how deformation resistance evolves throughout loading. This distinction is important for O-ring seals because progressive stiffness changes can influence contact-pressure retention during service. Because the descriptors discussed thus far retain absolute mechanical magnitude, direct comparison remains influenced by intrinsic differences in stiffness and hardness among the materials. To isolate differences in deformation behavior, Figure 6 presents normalized force-displacement curves at the lowest and highest hydrogen exposure pressures (800 and 7000 psi). In this representation, variations between curves primarily reflect differences in deformation trajectory rather than absolute load-bearing capacity.

At 800 psi, the three elastomers exhibited distinct curve shapes during the intermediate deformation stages. FKM showed a steeper early-stage response, indicating greater resistance to deformation during the initial loading region. In contrast, EPDM displayed a more gradual force buildup over a wider displacement range, corresponding to a softer deformation response. NBR exhibited intermediate behavior between the two materials.

At 7000 psi, the normalized responses of NBR and FKM became more closely aligned across most of the loading range, while EPDM continued to exhibit a broader and more gradual deformation progression. Despite differences in absolute mechanical properties observed in previous sections, the normalized curves indicate that all materials maintained generally continuous nonlinear loading behavior without abrupt instability prior to peak force. While the preceding descriptor-based analysis provides insight into individual aspects of tensile-response evolution, hydrogen-induced degradation in elastomeric materials emerges through coupled interactions among stiffness redistribution, deformation progression, and energy absorption behavior. Consequently, multivariate statistical analysis and machine-learning-assisted interpretation were applied to identify latent response relationships and material-specific degradation pathways that may not be evident from isolated mechanical descriptors alone.

### 3.2. Multivariate Statistical and Machine-Learning-Based Degradation Analysis

#### Correlation Analysis of Mechanical Degradation Descriptors

**Figure 7:**
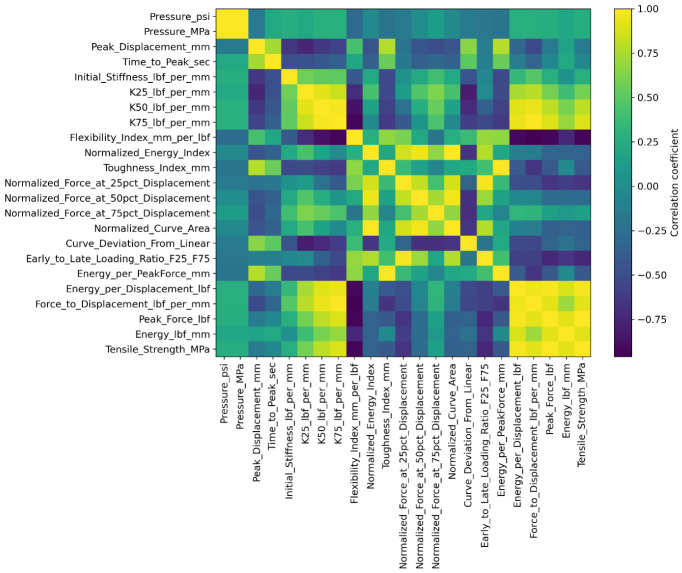
Correlation matrix of experimentally measured and derived mechanical descriptors for hydrogen-exposed elastomeric materials. The heatmap illustrates relationships among stiffness-related, energy-based, deformation, and normalized response parameters across all investigated materials and pressure conditions. Positive correlations indicate coupled mechanical evolution, whereas negative correlations reflect competing deformation and stiffness behaviors.

The scalar descriptors examined above are not independent of one another: peak force, energy absorption, and stiffness all couple to the same underlying load–deformation behavior, while flexibility-related metrics necessarily move in the opposite direction. To make these couplings explicit before proceeding to dimensionality reduction, Figure 7 shows the Pearson correlation matrix among all extracted descriptors across the full set of materials and pressures.

Figure 7 reveals the presence of several strongly correlated descriptor groups associated with load-bearing behavior and deformation resistance. In particular, peak force, energy absorption, and secant stiffness descriptors evaluated at different stages of deformation (K25, K50, and K75) exhibit strong positive correlations with one another, indicating that these quantities evolve consistently as the mechanical resistance of the elastomer increases. Similarly, the energy-per-displacement and force-to-displacement ratios show a close association with stiffness metrics, demonstrating the coupled nature of deformation resistance and energy storage capability during tensile loading.

In contrast, flexibility-related descriptors demonstrate generally negative correlations with stiffness- and force-dominated parameters. The flexibility index and peak displacement metrics exhibit inverse trends relative to stiffness- and force-related quantities, showing that materials capable of larger deformation tend to exhibit lower resistance to tensile loading. This behavior is consistent with the competing relationship between compliance and structural rigidity in elastomeric systems subjected to hydrogen exposure.

The normalized force descriptors evaluated at 25%, 50%, and 75% displacement display moderate-to-strong positive correlations with energy-based metrics and secant stiffness parameters, demonstrating that the overall deformation pathway remains closely linked to progressive load development throughout tensile deformation. Furthermore, the strong intercorrelation among K25, K50, and K75 indicates that the relative stiffness evolution during loading remains internally consistent despite differences in material type and hydrogen pressure.

**Figure 8:**
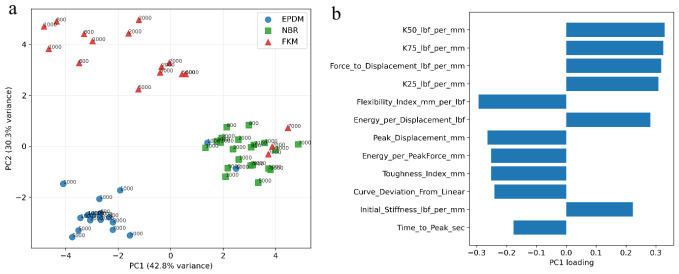
(**a**) Principal component analysis (PCA) score map showing clustering behavior of EPDM, NBR, and FKM specimens based on experimentally measured and derived mechanical descriptors under different hydrogen pressure conditions. PC1 and PC2 explain 42.8% and 30.3% of the total variance, respectively. (**b**) Dominant PC1 loading contributions illustrating the relative influence of stiffness-, deformation-, and energy-related descriptors on the primary variance direction governing material separation.

The correlation matrix also highlights descriptors exhibiting relatively weak or mixed correlations, particularly those associated with curve-deviation and nonlinear deformation behavior. These weaker relationships suggest that certain aspects of the mechanical response evolve independently of conventional strength- and stiffness-related metrics and may therefore provide complementary information regarding material-dependent deformation behavior. At the same time, the strong intercorrelations among many descriptors indicate that the overall feature space contains substantial redundancy and can be represented using a smaller number of orthogonal variables without significant loss of information. Principal component analysis (PCA) was therefore applied to the standardized descriptor matrix to identify the dominant variance directions. Figure 8a presents the resulting two-dimensional score map, while Figure 8b shows the PC1 loading distribution used to interpret the mechanical factors governing material separation.

The PCA score map in Figure 8a demonstrates clear clustering behavior among the investigated elastomer families. EPDM, NBR, and FKM occupy distinct regions within the PC1–PC2 space, indicating that each material exhibits a characteristic mechanical response pattern under hydrogen exposure. The separation along PC1, which explains 42.8% of the total variance, is primarily associated with differences in stiffness-related and force-dominated mechanical behavior. In contrast, PC2 (30.3% variance) captures secondary variations associated with deformation progression and nonlinear response evolution. Together, PC1 and PC2 account for 73.1% of the total variance in the descriptor dataset. The remaining 26.9% is distributed among higher-order principal components and likely reflects more subtle variations associated with nonlinear curve-shape descriptors, specimen-to-specimen variability, pressure-specific response redistribution, and interactions among stiffness, deformation, and energy-related features that are not fully captured by the first two principal components. Although these higher-order components individually explain smaller fractions of variance, they may still contain information relevant to hydrogen-induced response evolution. However, because their contributions are distributed across multiple orthogonal dimensions and do not produce clear material separation, the dominant degradation trends are adequately represented by the first two principal components used in the present analysis.

The PCA score map demonstrates that material identity remains the dominant source of variation within the descriptor dataset. This conclusion is supported by the observation that the separation among the EPDM, NBR, and FKM clusters is generally larger than the pressure-induced spread within each individual material group. In particular, the three elastomer families remain well separated throughout the lower and intermediate pressure range (800–2000 psi), indicating that intrinsic material characteristics such as polymer chemistry, polarity, network structure, and deformation behavior exert a stronger influence on the descriptor space than hydrogen pressure alone. At higher pressures, especially near 7000 psi, pressure-dependent effects become more pronounced, most notably for FKM, which exhibits larger movement within the PCA space and stronger changes in stiffness- and force-related descriptors. However, the material-specific clusters remain distinguishable even at the highest investigated pressure, suggesting that hydrogen pressure primarily modifies material-specific response trajectories rather than overriding the influence of intrinsic material characteristics.

Although individual pressure conditions produce localized movement within the PCA space, the three elastomer families remain largely separated, indicating that intrinsic material characteristics exert a stronger influence on mechanical response evolution than hydrogen pressure alone. The broader spread observed for FKM suggests greater sensitivity to pressure-dependent response redistribution, whereas the tighter NBR cluster indicates comparatively stable descriptor behavior across the investigated conditions. EPDM occupies an intermediate region, characterized by greater dispersion than NBR but less directional separation than FKM, reflecting the more heterogeneous response trends observed in the descriptor analysis.

The PC1 loading distribution shown in Figure 8b identifies the descriptors contributing most strongly to the primary variance direction. Secant stiffness metrics (K25, K50, and K75), force-to-displacement ratio, and energy-per-displacement parameters exhibit the largest positive loadings, indicating that stiffness evolution and deformation resistance are the dominant factors governing material separation. Conversely, flexibility-related descriptors, particularly flexibility index and peak displacement, contribute in the opposite direction, reflecting the trade-off between compliance and stiffness observed throughout the mechanical dataset. These loading patterns explain the clustering structure in Figure 8a and demonstrate that the PCA separation is governed primarily by differences in stiffness progression and energy-related response, rather than by pressure alone.

**Figure 9:**
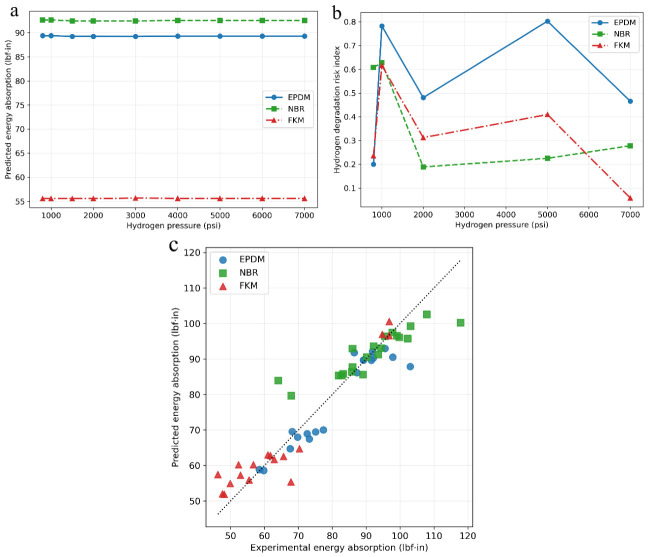
Machine-learning-assisted prediction and comparative response assessment of elastomer mechanical behavior under hydrogen exposure. (**a**) Predicted energy-absorption trends across hydrogen pressure conditions obtained using the Random Forest regression framework. (**b**) Pressure-dependent evolution of the Composite Hydrogen Response Index (CHRI), calculated from standardized stiffness, defect-density, and defect-area-fraction descriptors to provide an integrated representation of the combined mechanical and optical response state. (**c**) Comparison between experimentally measured and LOOCV-predicted energy absorption values shows overall predictive agreement of the machine-learning framework.

To further evaluate the coupled mechanical degradation behavior of the investigated elastomers, a machine learning framework based on Random Forest regression was implemented using the experimentally measured and derived mechanical descriptors. The model was trained to identify relationships between hydrogen exposure conditions and energy absorption behavior while simultaneously providing comparative degradation assessment across EPDM, NBR, and FKM materials. The resulting predictive behavior and degradation assessment are presented in Figure 9.

Linear projection separates the three elastomer families cleanly along PC1, but the within-material pressure dependence of mechanical response is non-monotonic and therefore is not fully captured by a single linear component. To examine nonlinear descriptor-to-property relationships, a Random Forest regression model was trained to predict energy absorption from the complete descriptor set, with leave-one-out cross-validation (LOOCV) used to evaluate predictive agreement on held-out specimens. Figure 9 summarizes the resulting analysis: panel (a) shows the predicted energy-absorption response as a function of hydrogen pressure for each material, panel (b) presents the pressure-dependent evolution of the Composite Hydrogen Response Index (CHRI) defined in Section 2.6, and panel (c) compares experimentally measured and LOOCV-predicted energy-absorption values. Across the pooled three-material dataset, the LOOCV analysis produced an overall coefficient of determination of R^2^ = 0.888 and a root-mean-square error (RMSE) of 5.94 lbf·in for energy-absorption prediction across 57 analyzed specimens.

The pressure-dependent trends shown in Figure 9a demonstrate clear differences in response magnitude among the investigated elastomer systems. NBR consistently exhibited the highest predicted energy-absorption values across the investigated pressure range, followed by EPDM, whereas FKM maintained lower predicted values. The reported R^2^ and RMSE values should be interpreted as indicators of internal consistency within the present dataset, rather than as evidence of generalized predictive capability beyond the investigated materials and exposure conditions.

The CHRI trends shown in Figure 9b indicate distinct combined mechanical-optical response evolution among the investigated elastomer systems. For FKM, CHRI values were calculated only for pressure conditions with complete optical descriptor datasets (800, 1000, and 7000 psi); no interpolation or synthetic data generation was performed for the missing intermediate-pressure optical conditions. EPDM exhibited larger fluctuations in CHRI across the investigated pressure range, suggesting greater sensitivity of the combined response state to hydrogen exposure. NBR showed a progressive increase in CHRI at elevated pressures, reflecting the combined influence of increasing defect density, increasing defect area fraction, and changes in the stiffness-related descriptor incorporated within the index. FKM exhibited comparatively moderate CHRI variation, consistent with its more stable optical response and constrained deformation evolution under hydrogen exposure. Overall, the CHRI results highlight the value of integrating mechanical and optical descriptors within a unified response metric, revealing trends that are not fully captured by individual parameters alone.

Figure 9c shows good agreement between experimentally measured and LOOCV-predicted energy-absorption values across the investigated conditions. The relatively high predictive agreement indicates that the extracted descriptor set successfully captured major deformation-response characteristics associated with hydrogen exposure within the present dataset. However, because of the limited dataset size and experimentally constrained pressure conditions, the regression behavior should be interpreted primarily as a within-dataset consistency indicator rather than as evidence of generalized predictive capability beyond the investigated materials and exposure conditions.

Feature-importance analysis indicated that deformation-related and stiffness-related descriptors, particularly energy-per-displacement ratio, peak displacement, and energy-per-peak-force ratio, contributed strongly to the regression behavior within the Random Forest framework. It should be noted that several descriptors included in the predictor set are mathematically related to the target variable (energy absorption) because they are derived from the same force-displacement response. Accordingly, the feature-importance rankings should be interpreted primarily as measures of statistical association and predictive contribution within the present dataset, rather than as direct evidence of independent physical causality. While the identified descriptors remain useful for characterizing comparative response evolution, the feature-importance analysis alone does not establish the underlying mechanisms governing hydrogen-induced degradation. These findings are consistent with the PCA and correlation analyses, which similarly identified coupled stiffness-, energy-, and deformation-related descriptors as dominant contributors governing the observed multivariate mechanical response evolution.

**Figure 10:**
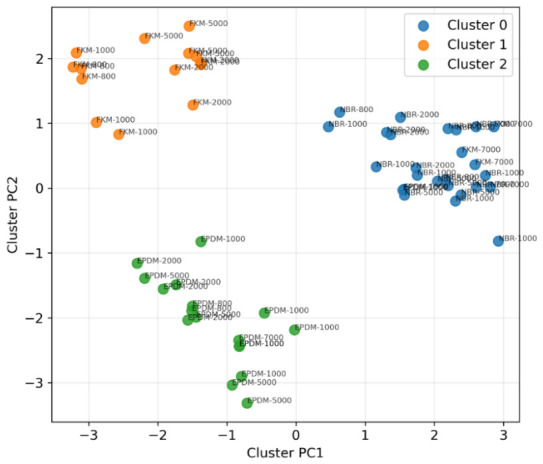
Unsupervised clustering analysis of hydrogen-induced mechanical degradation behavior based on PCA-transformed mechanical descriptors. Distinct cluster formation was observed for EPDM, NBR, and FKM specimens, indicating material-specific degradation pathways under high-pressure hydrogen exposure.

Taken together, the machine learning results demonstrate that hydrogen-induced response behavior can be differentiated using combined stiffness-, deformation-, and energy-related descriptors. While the PCA score map (Figure 8) and the supervised regression model (Figure 9) both indicated that material identity dominates the response distribution, neither directly tested whether the data partition naturally into discrete groups within the descriptor space. To address this, k-means clustering was applied to the PCA-reduced feature set without using material labels. Figure 10 presents the resulting cluster assignments together with the actual material identities, allowing direct assessment of whether the unsupervised partitioning recovers the material-dependent structure.

The clustering map demonstrates the formation of three dominant degradation-response regions corresponding primarily to EPDM, NBR, and FKM mechanical behavior. FKM specimens are concentrated within the upper-left region of the PCA space (Cluster 1), while EPDM specimens occupy the lower-left region (Cluster 2). In contrast, NBR specimens are primarily distributed within the positive PC1 region (Cluster 0). The clear separation between clusters indicates that each elastomer family exhibits a distinct hydrogen-induced degradation pathway, characterized by different combinations of stiffness evolution, deformation resistance, flexibility retention, and energy absorption behavior.

The compact clustering behavior observed for NBR suggests stable and consistent mechanical response characteristics across the investigated pressure range. EPDM exhibits broader vertical dispersion within the PCA space, demonstrating greater variability in pressure-dependent deformation behavior and mechanical instabilities. FKM demonstrates separation primarily along the PC2 direction, revealing that nonlinear deformation evolution and response redistribution contribute strongly to its hydrogen degradation behavior.

The clustering analysis supports the PCA and machine-learning results by demonstrating that the investigated elastomer systems follow distinct mechanical-response trajectories rather than a single universal response pattern. Because the present dataset remains relatively limited in size and was obtained under controlled laboratory conditions, the machine-learning results should be interpreted primarily as an exploratory within-dataset analysis rather than as a generalized predictive framework applicable to all hydrogen–elastomer systems.

An interesting contrast emerges between the mechanical and surface-state behavior of FKM. The bulk mechanical descriptors place FKM in a clearly separated cluster from EPDM and NBR (Figure 10) and show the strongest pressure sensitivity of the three materials, particularly at 7000 psi. In contrast, the optical defect metrics in the following section indicate that FKM exhibits the smallest pressure-dependent changes in surface state. Taken together, this pattern is consistent with a scenario in which the dominant hydrogen-induced response in FKM is internal—potentially involving pressure-driven changes in chain mobility and free-volume-related deformation behavior rather than surface-localized damage—whereas in NBR, hydrogen exposure produces a more visible surface signature. This interpretation is offered tentatively and would require complementary subsurface characterization (e.g., DMA, free-volume probes) for confirmation, but it is internally consistent across all three analysis branches presented in this study.

The mechanical analyses presented thus far characterize hydrogen-induced degradation through changes in stiffness evolution, deformation progression, and energy absorption behavior. However, degradation processes may also manifest through pressure-dependent changes in surface condition that are not fully captured by bulk mechanical measurements alone. Accordingly, the next section examines optical surface-state evolution using image-based defect quantification to determine whether the mechanical trends identified above are accompanied by corresponding changes in surface morphology.

### 3.3. Optical Surface Damage Evolution and Quantitative Defect Analysis

**Figure 11:**
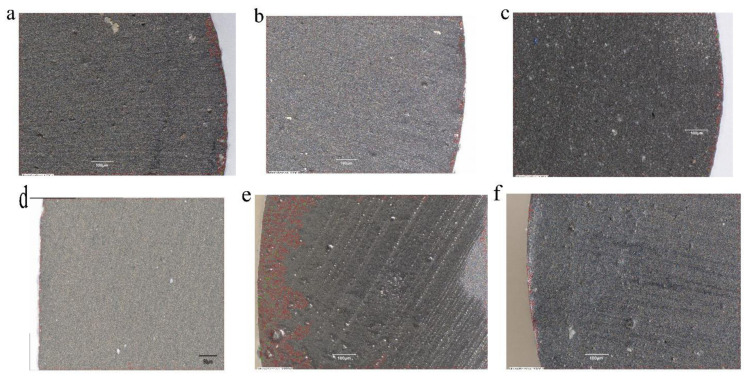
Representative optical surface images with automated segmentation overlays for EPDM, NBR, and FKM elastomer specimens following hydrogen exposure at 1000 psi (**a**–**c**) and 7000 psi (**d**–**f**). Panels (**a**,**d**) correspond to EPDM, panels (**b**,**e**) correspond to NBR, and panels (**c**,**f**) correspond to FKM. Red overlay regions indicate segmented surface defect features identified through image-processing-based contrast analysis. All images were acquired under consistent optical magnification and illumination conditions.

The mechanical analysis above probes the bulk load-deformation behavior of the elastomer network. To examine whether hydrogen exposure also alters the specimen surface in a way that could affect sealing performance, the contact-side surfaces of representative specimens were imaged under brightfield optical microscopy under identical illumination and magnification. Figure 11 shows representative micrographs together with the binary segmentation masks obtained from the automated image-processing workflow [20,21]; these masks form the basis for the quantitative optical metrics reported in Table 4 and the subsequent analysis.

Optical defect quantification was performed using a consistent automated image-processing workflow. Images were first converted to grayscale, and contrast-limited adaptive histogram equalization (CLAHE) was applied to reduce local illumination variation and improve contrast of surface features. A black-hat morphological operation was then used to enhance localized dark features relative to the surrounding background. Segmentation was performed using Otsu thresholding, which determines the threshold objectively from the image intensity distribution rather than through manual image-by-image adjustment. Morphological opening and closing were subsequently applied to remove isolated noise and smooth segmented regions. Connected-component/contour analysis was then used to extract individual surface features. Object-level filtering based on area, circularity, aspect ratio, and border exclusion was applied to reduce contributions from image noise, scale-bar regions, borders, and elongated texture features.

Because commercial elastomer surfaces can contain original roughness, manufacturing texture, and handling-related marks, the segmented regions should be interpreted as comparative optical defect indicators rather than as definitive proof of hydrogen-induced damage mechanisms. Representative binary masks and overlay images were visually compared with the original optical micrographs to verify that the segmented regions corresponded to observable surface features. The same processing workflow and filtering criteria were applied consistently across all materials and pressure conditions to minimize operator bias and enable relative comparison.

Values are reported as mean ± standard deviation obtained from automated optical image segmentation and connected-component-based defect analysis. Percentage changes were calculated relative to the lowest-pressure condition for each polymer. P90 defect size represents the 90th percentile equivalent defect diameter and was used to characterize larger surface defect populations. Optical surface characterization was performed at five pressure conditions for EPDM and NBR (800, 1000, 2000, 6000, and 7000 psi), whereas FKM optical characterization was limited to 800, 1000, and 7000 psi because of specimen-availability constraints during the optical imaging phase. Consequently, direct pressure-by-pressure optical comparison between FKM and the other two elastomer systems at intermediate pressure conditions is not possible within the present dataset.

**Figure 12:**
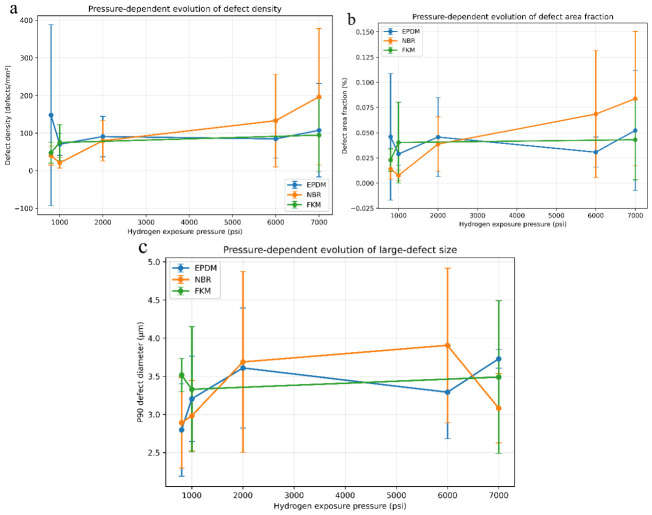
Pressure-dependent evolution of quantitative optical defect metrics for EPDM, NBR, and FKM elastomers following hydrogen exposure. (**a**) Defect density, (**b**) defect area fraction, and (**c**) P90 defect size obtained from automated image-segmentation-based optical analysis. Error bars represent the mean ± standard deviation calculated from multiple analyzed optical fields of view. The results indicate material-dependent and non-monotonic surface degradation behavior under increasing hydrogen exposure pressure conditions.

Table 4 summarizes the quantitative optical defect metrics obtained from automated image-segmentation analysis, including defect density, defect area fraction, and P90 defect size for EPDM, FKM, and NBR following hydrogen exposure. Overall, the results indicate distinct material-dependent surface-state evolution across the investigated elastomer systems. NBR exhibited the strongest increases in defect density and defect area fraction at elevated pressures, whereas FKM maintained comparatively smaller changes in the optical descriptors. EPDM showed more heterogeneous and non-monotonic behavior across the investigated pressure conditions. The detailed pressure-dependent evolution of these metrics is further presented in Figure 12.

Collectively, the evolution of defect density, area fraction, and P90 defect size suggests that hydrogen exposure influences both the frequency and spatial extent of surface damage features. The differing responses observed among NBR, EPDM, and FKM are consistent with material-dependent hydrogen transport and deformation behavior; however, the optical measurements alone do not permit the definitive identification of the underlying degradation mechanisms.

The relatively large standard deviations observed for several conditions indicate that surface degradation evolved heterogeneously across the analyzed regions rather than uniformly across the specimen surface. Consequently, the optical descriptors should be interpreted as comparative indicators of surface-state evolution rather than direct evidence of subsurface structural damage. An important observation is that NBR exhibited the largest increases in defect density and defect area fraction while simultaneously maintaining a comparatively high load-bearing capability and stiffness-related response. This apparent contrast suggests that the quantified optical features represent localized surface-state changes rather than extensive bulk mechanical degradation. The measured defect sizes remained small relative to the 3.53 mm O-ring cross-sectional diameter, and the observed defect regions occupied only a limited fraction of the total load-bearing area. Consequently, the bulk elastomer network remained the dominant contributor to the tensile response measured in the present study. Nevertheless, under long-term service conditions involving cyclic loading, repeated decompression, thermal fluctuations, or environmental aging, such surface features could potentially act as stress-concentration sites and contribute to progressive damage accumulation. Evaluation of these long-term effects requires additional investigation beyond the scope of the present work.

To complement the quantitative optical metrics summarized in Table 4, Figure 11 presents representative optical micrographs and corresponding segmentation overlays for EPDM, NBR, and FKM specimens following hydrogen exposure under selected pressure conditions. The segmented regions provide a qualitative visualization of the pressure-dependent surface-state evolution identified through the automated image-processing workflow using established digital image-analysis procedures [20,21]. These observations support the comparison of relative surface-condition changes among materials and exposure conditions, and should be interpreted in conjunction with the quantitative metrics reported in Table 4 and Figure 12.

Visual inspection of the masks in Figure 11 suggests pressure-dependent and material-dependent differences in surface state, but a quantitative readout is required to compare these trends across the full pressure series. Figure 12 plots the three primary defect metrics extracted from the segmented masks—defect density, defect area fraction, and the 90th-percentile equivalent defect diameter (P90)—as a function of hydrogen exposure pressure for the three materials.

Among the investigated materials, NBR exhibited the strongest overall increase in both defect density and defect area fraction at elevated pressures, demonstrating progressively increased formation and accumulation of distributed surface defect features under high-pressure hydrogen exposure. The P90 defect size initially increased under intermediate pressure conditions before decreasing at 7000 psi, indicating that defect evolution may involve both localized defect growth and heterogeneous redistribution behavior rather than purely monotonic enlargement.

EPDM exhibited non-monotonic behavior across the investigated pressure conditions. Although moderate increases in defect size were observed at elevated pressures, the defect density and area fraction fluctuated between pressure conditions rather than increasing continuously. This behavior suggests a more heterogeneous surface-state evolution than that observed for NBR and indicates that multiple competing processes may contribute to the pressure-dependent response.

FKM demonstrated smaller overall changes in the quantified optical metrics relative to NBR. Although gradual increases in defect density and area fraction were observed with increasing pressure, the overall magnitude of change remained comparatively limited. The P90 defect size also remained relatively stable across the investigated pressure conditions, consistent with limited pressure-driven surface defect evolution under the current experimental conditions. The relatively large standard deviations observed for several conditions indicate that the surface degradation process was spatially heterogeneous rather than uniformly distributed across the analyzed regions. Since the present analysis relied primarily on optical surface characterization, the quantified trends should be interpreted as comparative indicators of surface damage evolution rather than direct evidence of subsurface structural degradation. Additional characterization techniques, such as SEM, DMA, sorption analysis, or free-volume characterization, may be required to further validate the underlying hydrogen-induced degradation mechanisms.

**Figure 13:**
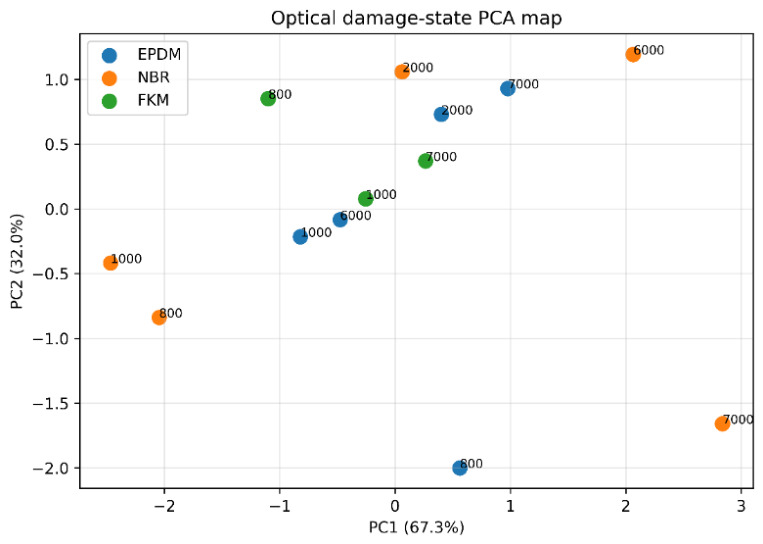
Principal component analysis (PCA) map constructed from the quantified optical defect metrics for EPDM, NBR, and FKM elastomers under different hydrogen exposure pressures. The PCA projection was generated using defect density, defect area fraction, and P90 defect size, which were obtained from automated optical image analysis. PC1 and PC2 explained 67.3% and 32.0% of the total variance, respectively. The clustering behavior indicates material-dependent and pressure-dependent differences in the optical damage-state evolution.

The three optical descriptors in Figure 12 are not independent because defect density, defect area fraction, and defect size evolve through related surface-state changes. Accordingly, Figure 13 presents a PCA projection constructed from the standardized optical metrics for all material-pressure conditions, providing an integrated representation of the quantified optical response. The first principal component (PC1) explained approximately 67.3% of the total variance and primarily captured the overall progression of surface-state evolution, whereas PC2 explained an additional 32.0% of the variance, which was associated with secondary variations in defect morphology. Together, the first two principal components captured nearly the entire variance of the analyzed optical dataset.

Among the investigated materials, NBR exhibited the largest spatial separation across pressure conditions, particularly at elevated pressures, indicating stronger pressure-dependent evolution of the quantified surface state. In contrast, FKM exhibited tighter clustering behavior, consistent with a comparatively stable optical response under increasing hydrogen exposure pressure. EPDM occupied an intermediate position with moderate dispersion between pressure conditions, reflecting the non-monotonic trends observed in the defect metrics. Because the FKM optical dataset included only three pressure conditions, compared with five conditions for EPDM and NBR, the apparent clustering behavior for FKM warrants cautious interpretation, rather than being considered a fully pressure-resolved trajectory equivalent to the other elastomer systems. The absence of intermediate-pressure optical measurements (2000 and 6000 psi) also limits assessment of whether the optical-response evolution of FKM is monotonic or non-monotonic across the investigated pressure range. Accordingly, the present dataset supports comparison of the overall magnitude of optical-response change between FKM and the other elastomer systems but does not permit detailed reconstruction of the complete pressure-dependent optical trajectory for FKM.

Overall, the PCA results demonstrate that the combined optical descriptors differentiate material-dependent surface-response behavior and provide a compact multivariate representation of pressure-dependent surface-state evolution. However, because the analysis is derived solely from optical surface metrics, the observed clustering patterns should not be interpreted as direct evidence for specific molecular degradation mechanisms. Additional complementary characterization techniques would be required to establish definitive structure-property relationships associated with hydrogen-induced degradation behavior.

## 4. Discussion, Limitations and Future Research Directions

### 4.1. Integrated Discussion

The combined mechanical and optical analyses reveal that hydrogen-induced response evolution differs substantially among the investigated elastomer systems and cannot be described by a single degradation pathway. NBR maintained high load-bearing capability and stiffness-related response while exhibiting the largest increases in optical defect density and area fraction, indicating that pronounced surface-state evolution can occur despite relatively stable bulk mechanical behavior. In contrast, EPDM exhibited greater variability and non-monotonic trends in both the mechanical and optical datasets, suggesting a more heterogeneous response to hydrogen exposure.

The non-monotonic behavior observed for EPDM may reflect the simultaneous influence of multiple competing hydrogen-induced processes rather than a single dominant degradation mechanism. Depending on hydrogen pressure, the relative contributions of hydrogen sorption, transient network relaxation, swelling-related deformation, decompression-induced internal stress development, and localized microstructural evolution may differ. As a result, mechanical descriptors and optical defect metrics do not necessarily evolve in a monotonic manner with increasing pressure. The present results therefore suggest that different mechanisms may dominate in different pressure ranges, although the available data do not permit direct identification of the governing molecular processes. Verification of this interpretation would require complementary characterization methods such as DMA, hydrogen sorption analysis, free-volume measurements, and spectroscopic techniques capable of probing pressure-dependent structural evolution.

The most notable contrast was observed for FKM. Mechanical descriptors, PCA clustering, and machine-learning analysis identified FKM as the material exhibiting the strongest pressure-dependent redistribution of mechanical response, particularly at 7000 psi, whereas the optical characterization showed comparatively limited changes in defect density and area fraction. This divergence suggests that hydrogen exposure influences FKM primarily through changes affecting bulk deformation behavior rather than extensive surface-localized damage. One possible explanation is that hydrogen exposure modifies internal deformation processes associated with molecular mobility, free-volume redistribution, or network-level chain dynamics within the fluorocarbon elastomer, thereby influencing stiffness evolution, energy absorption, and deformation progression without necessarily producing extensive surface damage detectable by optical microscopy. However, the present study does not provide direct evidence for these mechanisms, and the observed behavior should therefore be interpreted as a pressure-dependent mechanical response trend rather than a confirmed structural transformation. Future studies incorporating SEM, ATR-FTIR, DMA, hydrogen sorption analysis, free-volume characterization, and other complementary physicochemical techniques would be required to establish the underlying structure–property relationships.

Overall, the agreement between the mechanical and optical analyses demonstrates the value of combining multivariate mechanical characterization with quantitative surface assessment to evaluate hydrogen-induced degradation behavior in elastomeric sealing materials. While the preceding discussion provides insight into hydrogen-induced response evolution under tensile loading, practical sealing applications involve different loading conditions, which should be considered when interpreting the results.

Although elastomer seals primarily operate under compressive loading conditions in practical hydrogen systems, tensile characterization provides useful insight into hydrogen-induced changes in load-bearing capability, deformation resistance, stiffness evolution, and overall network integrity. These characteristics may indirectly influence sealing behavior by affecting contact-force development, deformation stability, and resistance to damage accumulation. However, tensile-response descriptors should not be interpreted as direct measures of sealing performance because practical seal functionality additionally depends on compression set, compression stress relaxation, contact-pressure retention, and leakage resistance. Accordingly, the present results provide a comparative assessment of hydrogen-induced mechanical response evolution, while future studies incorporating compression-based characterization and leakage testing would help establish stronger links between material degradation and actual sealing performance.

### 4.2. Limitations and Future Research Directions

The present study provides a comparative experimental and data-driven evaluation of hydrogen-induced mechanical and surface degradation behavior in EPDM, NBR, and FKM elastomeric O-ring materials under controlled high-pressure hydrogen exposure conditions. However, several limitations should be acknowledged when interpreting the results.

First, the investigation primarily focused on post-exposure tensile response and optical surface characterization following hydrogen aging under static isothermal conditions. Although the selected testing methodology enables comparative evaluation of pressure-dependent degradation behavior, the present experiments do not fully represent the complex cyclic thermo-mechanical loading conditions encountered in practical hydrogen sealing systems. In-service seal behavior may additionally involve pressure cycling, repeated decompression, dynamic contact loading, thermal fluctuations, and long-term viscoelastic relaxation phenomena that were not addressed in the current work.

Second, the optical image analysis performed in this study was based on automated, contrast-based segmentation of surface features observed under optical microscopy. While the resulting quantitative metrics provide useful indicators of surface degradation evolution, the analysis does not directly confirm subsurface void formation, crack propagation, filler debonding, or molecular-level structural degradation. Consequently, the segmented surface features are best regarded as comparative optical defect indicators.

In addition, optical microscopy is inherently limited to surface-visible features and does not provide direct information regarding nanoscale defects, subsurface voids, internal cavitation, crack depth, filler–matrix debonding, or other microstructural changes that may develop during hydrogen exposure. As a result, the absence of pronounced optical surface damage should not be interpreted as evidence that internal degradation processes are absent. Complementary characterization techniques, such as scanning electron microscopy (SEM), X-ray micro-computed tomography (µCT), free-volume characterization, and other high-resolution methods, would be required to evaluate degradation phenomena occurring below the optical-resolution limit or beneath the specimen surface.

Third, the present study focused primarily on force–displacement-based descriptor extraction using practical O-ring geometries rather than conventional stress–strain characterization. Although this approach provides mechanically relevant information for sealing applications, the spool-based loading configuration does not produce a uniform stress field and therefore limits direct comparison with standardized tensile-property datasets reported in the broader elastomer literature. Accordingly, stiffness and energy-absorption descriptors reported in this work should not be interpreted as geometry-independent material properties. Rather, they represent comparative mechanical-response indicators for the specific O-ring geometry and spool-loading configuration used in this study. Future work using complementary dumbbell-type tensile specimens or finite-element analysis of the O-ring loading configuration could help separate intrinsic material behavior from geometry-dependent effects.

In addition, the machine-learning-assisted analysis presented in this work was intended primarily as a comparative interpretation framework for the experimentally generated dataset rather than as a generalized predictive model applicable to all hydrogen–elastomer systems. Similarly, the proposed CHRI formulation represents a comparative descriptor-based response metric derived from the present experimental dataset and has not been validated as a quantitative predictor of sealing failure, leakage onset, or long-term service life.

Future work should incorporate complementary physicochemical characterization techniques to further investigate the molecular- and microstructural-level mechanisms that govern hydrogen-induced degradation behavior. In particular, dynamic mechanical analysis (DMA) would be valuable for evaluating time-dependent viscoelastic evolution, relaxation behavior, storage modulus changes, and damping characteristics following hydrogen exposure. ATR-FTIR spectroscopy and solid-state NMR could further clarify chemical, structural, and chain-mobility changes associated with hydrogen exposure.

Additional future studies involving scanning electron microscopy (SEM), free-volume characterization, sorption analysis, permeability measurements, and decompression-cycling experiments would further strengthen understanding of the coupled transport–mechanical degradation mechanisms governing elastomer performance in high-pressure hydrogen environments. Expansion of the current framework to include time-dependent aging conditions, cyclic loading environments, and larger experimental datasets may also improve the robustness of future machine-learning-assisted degradation assessment approaches.

An additional limitation is that an unexposed (0 psi) control condition was not included in the experimental matrix. As a result, the present study evaluates relative pressure-dependent response evolution among hydrogen-exposed specimens but does not directly quantify the absolute magnitude of degradation relative to the as-received material condition.

The present study was also limited to room-temperature hydrogen-aging exposures and subsequent characterization. In practical hydrogen storage, transportation, and refueling systems, elastomer seals may experience a substantially broader temperature range, including sub-ambient and elevated-temperature conditions. Because temperature influences hydrogen diffusion, sorption behavior, desorption kinetics, viscoelastic response, chain mobility, swelling, and stiffness evolution, the pressure-dependent trends reported in this study should not be assumed to remain unchanged outside the investigated temperature regime. Future studies incorporating combined pressure-temperature exposure protocols would help establish the temperature dependence of hydrogen-induced response evolution and improve the applicability of laboratory observations to real service environments.

The present investigation employed a single decompression rate (approximately 70 bar min^−1^) for all hydrogen-aging experiments. Because decompression history can influence hydrogen-release kinetics and decompression-induced damage mechanisms, the observed response evolution may differ under alternative industrial decompression scenarios. Future studies examining multiple decompression rates would help establish the sensitivity of elastomer degradation behavior to hydrogen-release conditions.

Because testing was conducted shortly after depressurization, the present dataset does not distinguish between reversible mechanical changes associated with residual dissolved hydrogen and more persistent hydrogen-induced microstructural alterations. Future studies incorporating controlled degassing intervals, hydrogen-content measurements, or time-dependent post-exposure characterization would help separate transient plasticization effects from longer-term structural evolution.

A further limitation arises from the use of commercial elastomer compounds for which detailed formulation information is not available. Because filler content, curing chemistry, crosslink density, plasticizer concentration, and other formulation variables can significantly influence hydrogen compatibility, the quantitative results obtained in this study should be interpreted as specific to the investigated materials and not as universally representative of all formulations within the EPDM, NBR, or FKM elastomer families.

## 5. Conclusions

This study presented a comparative experimental and data-driven investigation of hydrogen-induced mechanical and surface degradation behavior in EPDM, NBR, and FKM elastomeric O-ring materials following prolonged high-pressure hydrogen exposure under controlled isothermal conditions.

Analysis of the extracted mechanical features demonstrated that hydrogen exposure influences not only peak mechanical response but also stiffness evolution, deformation progression, and energy-absorption behavior throughout tensile loading. Correlation analysis, principal component analysis, clustering analysis, and machine-learning-assisted interpretation collectively showed that degradation behavior emerges from coupled multivariate interactions rather than from isolated single-parameter changes. The combined mechanical and optical datasets further revealed that the relative contributions of bulk mechanical evolution and surface-state change differ among elastomer systems, highlighting the importance of evaluating hydrogen-induced degradation using multiple complementary response domains.

A key contribution of this work is the integration of whole-curve mechanical descriptor extraction, quantitative optical characterization, multivariate statistical analysis, and machine-learning-assisted interpretation within a unified assessment framework. The agreement among independent analytical approaches—including correlation analysis, PCA, clustering behavior, and Random Forest regression—suggests that meaningful degradation-related response patterns can be identified from experimentally measured force–displacement behavior beyond conventional peak-response metrics alone. These results illustrate the value of combining data-driven analysis with experimental characterization to improve understanding of pressure-dependent degradation behavior in hydrogen-exposed elastomeric materials.

The framework developed in this study provides a foundation for future hydrogen-compatibility assessment methodologies for elastomer sealing materials used in high-pressure hydrogen infrastructure systems. Future investigations incorporating dynamic mechanical analysis (DMA), spectroscopic characterization, free-volume measurements, sorption analysis, and cyclic hydrogen exposure conditions may provide additional insights into the structure–property relationships associated with hydrogen-induced degradation and support the development of more predictive material-selection and seal-performance assessment strategies.

## Figures and Tables

**Table 1:** Material specifications and geometric properties of the elastomer O-ring specimens used in this study.

Property	EPDM	NBR (Buna-N)	FKM (Viton^®^)
Material type	EPDM rubber	Nitrile rubber	Fluorocarbon elastomer
Typical polarity	Non-polar	Moderately polar	Fluorinated/polar
Standard	ASTM D2000/SAE AS568	ASTM D2000/SAE AS568	SAE AS568
Hardness	70 Shore A	70 Shore A	60 Shore A
Inner diameter	0.984 in (25.0 mm)	0.984 in (25.0 mm)	0.984 in (25.0 mm)
Outer diameter	1.262 in (32.05 mm)	1.262 in (32.05 mm)	1.262 in (32.05 mm)
Cross-section diameter	0.139 in (3.53 mm)	0.139 in (3.53 mm)	0.139 in (3.53 mm)
Dash size	AS568-214	AS568-214	AS568-214
Representative composition	Ethylene–propylene–diene elastomer	Acrylonitrile–butadiene elastomer	VDF/HFP-based fluorocarbon elastomer
Relative gas transport tendency	High	Moderate	Low
Typical mechanical behavior	Flexible/high extensibility	Higher stiffness/moderate extensibility	Chemically resistant/constrained network

**Table 2:** Summary of the hydrogen exposure conditions and experimental parameters used in this study.

Parameter	Value
Materials	EPDM, NBR, and FKM O-rings
Specimen geometry	AS568-214 O-rings
Cross-section diameter	0.139 in (3.53 mm)
Hydrogen aging pressure	800–7000 psi
Aging duration	192 h
Aging temperature	Room temperature
Hydrogen purity	High-purity H_2_
Tensile loading mode	Displacement-controlled
Number of replicates	*n* ≥ 3 per condition

**Table 3:** Summary of mechanical descriptors extracted from the force–displacement response. Detailed mathematical formulations are provided in Ref. [15].

Descriptor Category	Descriptor	Physical Interpretation
Conventional Response	Peak Force	Maximum load sustained before rupture
Conventional Response	Peak Displacement	Displacement corresponding to peak force
Energy Response	Energy Absorption	Mechanical work absorbed up to peak force
Stiffness Evolution	K25	Secant stiffness at 25% of peak displacement (early-stage deformation resistance)
Stiffness Evolution	K50	Secant stiffness at 50% of peak displacement (intermediate-stage deformation resistance)
Stiffness Evolution	K75	Secant stiffness at 75% of peak displacement (late-stage deformation resistance)
Normalized Response	Force–Displacement Ratio	Relative resistance to deformation
Normalized Response	Energy–Displacement Ratio	Energy absorbed per unit displacement
Flexibility Metrics	Flexibility Index	Relative deformation capability during loading
Curve Evolution	Normalized Curve-Shape Descriptors	Indicators of nonlinear response evolution independent of absolute force magnitude
Response Evolution	Stiffness-Progression Metrics	Quantify changes in stiffness across different deformation stages
Response Evolution	Curve-Deviation Metrics	Characterize departures from idealized response behavior

**Table 4:** Quantitative optical defect metrics obtained from surface image analysis following high-pressure hydrogen exposure.

Polymer	Pressure (psi)	Defect Density (Defects/mm^2^)	Area Fraction (%)	P90 Defect Size (µm)	% Change Density	% Change Area Fraction	% Change P90 Size
EPDM	800	147.67 ± 240.36	0.0459 ± 0.0628	2.80 ± 0.60	Baseline	Baseline	Baseline
EPDM	1000	70.27 ± 28.95	0.0289 ± 0.0112	3.21 ± 0.56	−52.40%	−37.10%	14.60%
EPDM	2000	90.83 ± 53.47	0.0456 ± 0.0393	3.61 ± 0.79	−38.50%	−0.70%	29.00%
EPDM	6000	84.62 ± 51.08	0.0307 ± 0.0150	3.29 ± 0.61	−42.70%	−33.20%	17.70%
EPDM	7000	107.59 ± 124.13	0.0522 ± 0.0596	3.73 ± 0.12	−27.10%	13.60%	33.30%
FKM	800	47.95 ± 27.46	0.0227 ± 0.0112	3.52 ± 0.22	Baseline	Baseline	Baseline
FKM	1000	74.76 ± 47.48	0.0402 ± 0.0401	3.33 ± 0.82	55.90%	76.80%	−5.30%
FKM	7000	94.69 ± 97.20	0.0429 ± 0.0397	3.49 ± 1.00	97.50%	88.80%	−0.80%
NBR	800	40.06 ± 25.19	0.0140 ± 0.0101	2.89 ± 0.59	Baseline	Baseline	Baseline
NBR	1000	21.23 ± 14.20	0.0076 ± 0.0051	2.98 ± 0.46	−47.00%	−45.90%	3.20%
NBR	2000	79.61 ± 53.76	0.0386 ± 0.0270	3.69 ± 1.18	98.70%	175.90%	27.60%
NBR	6000	133.07 ± 123.08	0.0685 ± 0.0632	3.91 ± 1.01	232.20%	388.90%	35.10%
NBR	7000	196.66 ± 181.27	0.0838 ± 0.0666	3.08 ± 0.45	390.90%	498.70%	6.60%

## Data Availability

The raw data supporting the conclusions of this article will be made available by the authors on request.

## Supplementary Materials

The following supporting information can be downloaded at https://www.mdpi.com/article/10.3390/polym18131570/s1, File S1: Mathematical Definitions of Core Mechanical Descriptors, including equations and definitions for energy absorption, secant stiffness (K25, K50, K75), late-stage instability, normalization procedures, peak force, peak displacement, force–displacement ratio (FDR), flexibility index (FI), and statistical metrics used in this study [19].
