# Supplementary material for: Data-Driven Multivariate Characterization of Hydrogen-Induced Response Evolution in EPDM, NBR, and FKM Elastomers
Source: Polymers (Basel). 2026 Jun 24;18(13):1570. doi: 10.3390/polym18131570 (PMC13364005; doi:10.3390/polym18131570)
Supplement: Supplementary file 1 [file polymers-18-01570-s001.zip › polymers-4370884-supplementary.pdf]

Mathematical Definitions of Core Mechanical Descriptors

To facilitate reproducibility, the principal mechanical descriptors used in this study are briefly summarized below. Detailed implementation procedures are available in Ref. [19].

1. **Energy absorption**

The mechanical energy absorbed during deformation was estimated as the area under the force–displacement curve up to the point of peak force. This quantity represents the work done on the specimen during loading and was calculated using numerical integration:

$$E = \int_0^{\delta_{\max}} F(\delta)\, d\delta$$

where $F(\delta)$ is the measured force and $\delta_{\max}$ is the displacement at peak force. Numerical integration was performed using the trapezoidal rule. The resulting energy values are reported in units of lbf·in and provide a resistance to loading and ability to absorb mechanical work prior to rupture.

2. **Secant stiffness**

To characterize the stiffness of the elastomer under finite displacement, secant stiffness values were calculated at specified displacement levels. Secant stiffness was defined as:

$$k_{secant} = \frac{F}{\delta}$$

where F is the force corresponding to a given displacement δ. In this study, stiffness values were evaluated at fixed fractions of the peak displacement (e.g., 25% and 50% of $\delta_{\max}$) using interpolation. This approach provides a stable and reproducible measure of stiffness that is less sensitive to local fluctuations compared to differential (tangent) stiffness.

3. Late-Stage Instability

$$I_{LS} = \sqrt{\frac{1}{n-1}\sum_{i=1}^{n}(F_i - \bar{F})^2}$$

where F_i represents the force values within the final 15% of the loading segment prior to peak force.

4. Normalization Equations

$$F_{norm} = \frac{F}{F_{peak}}$$

$$d_{norm} = \frac{d}{d_{peak}}$$

$$t_{norm} = \frac{t}{t_{peak}}$$

where:

F= measured force

d= measured displacement

t= measured time

F_{peak}= maximum force before rupture

d_{peak}= displacement at maximum force

t_{peak}= time corresponding to maximum force

5. Statistical Definitions

For a set of n replicate measurements:

Mean:

$$\bar{x} = \frac{1}{n}\sum_{i=1}^{n} x_i$$

Standard deviation:

$$s = \sqrt{\frac{1}{n-1}\sum_{i=1}^{n}(x_i - \bar{x})^2}$$

6. Peak Force

$$F_{peak} = \max(F)$$

where F is the measured tensile force.

7. Peak Displacement

$$d_{peak} = d(F_{peak})$$

where d_{peak} is the displacement corresponding to the maximum measured force.

8. K25 Secant Stiffness

$$K_{25} = \frac{F_{25}}{0.25\, d_{peak}}$$

where $F_{25}$is the force corresponding to 25% of peak displacement.

9. K50 Secant Stiffness

$$K_{50} = \frac{F_{50}}{0.50\, d_{peak}}$$

where $F_{50}$is the force corresponding to 50% of peak displacement.

10. K75 Secant Stiffness

$$K_{75} = \frac{F_{75}}{0.75\, d_{peak}}$$

where $F_{75}$is the force corresponding to 75% of peak displacement.

11. Force–Displacement Ratio (FDR)

$$FDR = \frac{F_{peak}}{d_{peak}}$$

This descriptor represents overall resistance to deformation.

12. Flexibility Index (FI)

$$FI = \frac{d_{peak}}{F_{peak}}$$

Higher values indicate greater deformation capability relative to load-bearing resistance.
